# Advanced conditioning of lignocellulosic wastes through imidazolium salts and ultrasound energy

**DOI:** 10.1098/rsos.250332

**Published:** 2025-10-08

**Authors:** Julieta Alvarez, M. Soledad Vela Gurovic, Gustavo F. Silbestri

**Affiliations:** ^1^ INQUISUR, Departamento de Química, Universidad Nacional del Sur (UNS)-CONICET, Bahía Blanca B8000CPB, Argentina; ^2^ CERZOS, Universidad Nacional del Sur - CONICET, Bahía Blanca , B8000FWB, Argentina

**Keywords:** lignocellulosic wastes, imidazolium salts, ultrasound energy

## Abstract

The concept of converting lignocellulosic waste into valuable products represents an innovative strategy that could yield significant economic benefits. Three water-soluble imidazolium salts were investigated for conditioning sunflower seed husks. Treatment conditions, including temperature and ultrasound (US), did not alter the chemical structure of the lignocellulosic residue, as confirmed by Fourier transform infrared analysis. Although US did not modify the functional groups, it did facilitate growth of the lignocellulose degrader *Streptomyces albus* CAS922—probably by causing rupture of fibres and plant cells. Additionally, the study on the reuse of aqueous solutions demonstrated that the salt remains effective for up to five consecutive cycles, exhibiting high stability to both temperature and US without any loss in concentration. The treatment discussed here could be appealing for the production of industrial products through biological processes.

## 1. Introduction

The growth of the global population increases the demand for agricultural production, leading to a rise in waste generation. The improper disposal of untreated agro-waste can severely impact the environment by contaminating both soil and water sources [1]. In 2022, a statistical study reported that 181.5 billion tonnes of lignocellulosic waste are produced worldwide, with only about 8.2 billion tonnes being used in various applications [1]. This suggests a substantial surplus of biomass residue that could be converted into value-added products, thereby reducing waste [1,2]. Lignocellulosic agro-residue comprises approximately 90% cellulose, hemicellulose and lignin, with the remaining 10% consisting of pectin and proteins. Additionally, it contains extractive compounds such as tannins, lipids, resins, steroids, terpenes, terpenoids, flavonoids and phenolic compounds [1,3,4]. The composition varies depending on the source but typically includes 40%–60% cellulose, 20%–40% hemicellulose and 10%–24% lignin [1]. As an example, in producing countries like Argentina, sunflower seed hulls make up 18%–20% of the seeds [5]. During the 2021/2022 season in Argentina, 1 968 907 hectares of sunflowers were planted, yielding 4 050 300 tonnes of seeds [6]. This resulted in approximately 810 060 tonnes of waste. This not only creates an environmental problem owing to its slow decomposition but also increases transportation costs owing to its low density. Sunflowers (*Helianthus annuus* L.) are native to North America and are a summer crop that is now cultivated in many temperate regions. They mature in 90–100 days. The sunflower head contains 1000–2000 individual flowers attached to the receptacle (stem) [5]. The seed hull constitutes 18%–20% of the processed seed and is made up of roughly 28.7% lignin, 31.3% cellulose and 25.5% hemicellulose, with the remainder comprising minerals, lipids, proteins and moisture [5]. Attempts to use this residue as fodder for cows and sheep have been unsuccessful owing to its high lignin content, which makes it unsuitable for this purpose. Burying the husks in the soil has also been tried, but this practice is risky without prior treatment owing to the presence of *Sclerotinia sclerotium*, a pathogenic fungus. As a result, the husks are often sold as firewood or used as bedding material for poultry and small mammals [7,8]. Research has also explored using this waste for mushroom cultivation [5] and pyrolytic treatment to produce oils [9]. Achieving a fully bio-based economy is vital for fulfilling sustainable development goals. In this context, lignocellulosic residues are valuable and cost-effective feedstocks for producing industrial products through biological processes. However, converting these materials efficiently poses challenges owing to their inherent resistance to bioprocessing [10]. They require preliminary treatment to alter their physicochemical properties, making their structural components more accessible. Among the various pretreatment methods, physical techniques show particular promise for the transformation of lignocellulosic residues. Gallego-García *et al.* [11] offer a thorough discussion on recent advancements in milling, extrusion, ultrasound (US) and microwave pretreatments. They analyse the mechanisms and application modes of these methods while addressing the key drawbacks and limitations associated with their industrial-scale implementation. It is well known that chemical and mechanical effects supplied by US come from cavitation. Growth and implosion of microbubbles in liquids will induce high temperatures and pressures inside such cavities, while shock waves at the interface and bulk liquid are largely responsible for enhanced mass and energy transfers [12]. Ravindran & Jaiswal conducted a comprehensive review on ultrasonic pre-treatment to break the complex polymerization network in biomass [13]. Cavitation generated by pulsating high-frequency ultrasonic waves penetrates polysaccharides, disrupting the network of cross-linked polymers and enhancing enzymatic degradation. For instance, Nakayama & Imai [14] reported an increase in cellulase adsorption when using US-pretreated kenaf leaves for enzymatic hydrolysis. Similarly, Li *et al.* [15] used a pretreatment combining ionic liquids (ILs) and US on rice straw. They found that FeCl_3_-assisted ultrasonic pretreatment improved acid hydrolysis of cellulose, leading to increased crystallinity.

On the other hand, ILs are organic salts that melt at temperatures below 100°C and are known for their excellent solvent properties. They are considered more environmentally friendly compared to conventional solvents because they have minimal vapour pressure and are flame retardant [16,17]. Brehm *et al.* [18] reported on the use of triazolium-based ILs as novel solvents for dissolving cellulose. They synthesized six ILs with 1,2,3-triazolium and 1,2,4-triazolium cations and found that the acetate-based ILs are room-temperature liquids capable of dissolving cellulose to levels comparable to imidazolium-based salts. Nuclear magnetic resonance (NMR) spectroscopy confirmed that cellulose does not degrade in these solutions. The study also demonstrated that cellulose solubility is more influenced by the basicity of the anion than by the type of cation. On the other hand, Seiler *et al.* [19] reported that N-butyl-N-methylpyrrolidinium hydroxide can dissolve up to 20 wt% of Avicel® cellulose at 25°C in a 50 wt% aqueous solution, making it the first effective pyrrolidinium-based salt for cellulose dissolution. Additionally, they employed turbidity as a practical and rapid method for assessing cellulose solubility.

Based on these observations, we propose to investigate the use of three imidazolium salts with water-soluble ligands, synthesized in our laboratory, to study the structural modification of lignocellulosic residue using US. To assess whether the salts and/or US have any biological detrimental effect on the husks, biological tests were conducted with the bacterium *Streptomyces albus* CAS922 [20]. The bacteria to be used produce ectoine, a substance of pharmaceutical interest owing to its beneficial properties. Ectoine reduces cellular stress and acts as a barrier to help recover damaged skin. It also provides protective effects against UV radiation and environmental pollution [21]. Additionally, we examined the recycling and reuse of the synthesized salts.

## 2. Material and methods

### 2.1. General procedures

All reactions were carried out under a dry nitrogen atmosphere by using Schlenk techniques [22]. Organic solvents were dried and distilled under nitrogen and degassed prior to use. Unless otherwise stated, reagents were obtained from commercial sources and used as received. ^1^H and ^13^C NMR spectra were recorded on a Bruker ARX 300 (300.1 MHz for ^1^H, 75.5 MHz for ^13^C) using D_2_O or dimethyl sulfoxide (DMSO)-d_6_ as solvents. ^1^H and ^13^C NMR spectra for each compound are shown in the electronic supplementary material. Ultraviolet-visible (UV–Vis) spectra were recorded in a Carey 60 version 2.0 instrument with a quartz 5 mL cell. Infrared spectra were collected on an attenuated total reflectance-Fourier transform infrared (ATR-FTIR) spectrometer Nicolet Nexus-470. A Cole Parmer 4710 series ultrasonic homogenizer operating at 20 kHz (600 W) with maximum nominal power of 187 W. The intensity of the US was adjusted by modifying the output level (1–4), and the duty cycle ranged from 10% to 80%. The duty cycle represents—the proportion of time during which US is emitted within a given period—essentially, the ratio of pulse duration to the total pulse interval [23]. This consists of an ultrasonic generator equipped with a probe that emits the sound vibration in the solution through a titanium alloy bar (5 mm diameter) dipped into the top of the liquid (image in the electronic supplementary material).

### 2.2. Sulfonate-imidazolium salts

The sulfonate-imidazolium salts **S1** and **S2** were synthesized in high yields by quaternization of the corresponding N-substituted imidazoles with 1,3-propanesultone according to the reported method [24,25]. **S3** was synthesized from imidazole and sodium−2-bromoethanesulfonate according to the reported method [26].

### 2.3. Ultrasonic irradiation

#### 2.3.1. Initial tests: crushed sunflower husks

The initial tests were conducted using crushed sunflower husks (powder). The vessel containing an aqueous solution of the salt (0.5% or 2.5%) and the sunflower husks was subjected to US treatment (power of 4 or 1, and duty cycle of 80% or 10%, respectively) for 5 min at a constant temperature of 80°C. To observe the effects of different instrumental conditions and salts on the sunflower husk powder suspension, biological tests were conducted with *S. albus* CAS922 to assess bacterial growth. The crushed sunflower husk suspension resulted in a highly compacted medium that prevented the growth of this soil bacterium. The whole sunflower shells were a better surface where this Streptomycete grew in contact with air. Also, untreated whole shells showed a predominance of fungal growth over the bacteria of interest.

#### 2.3.2. Final test: whole sunflower husks

**Figure 1.**
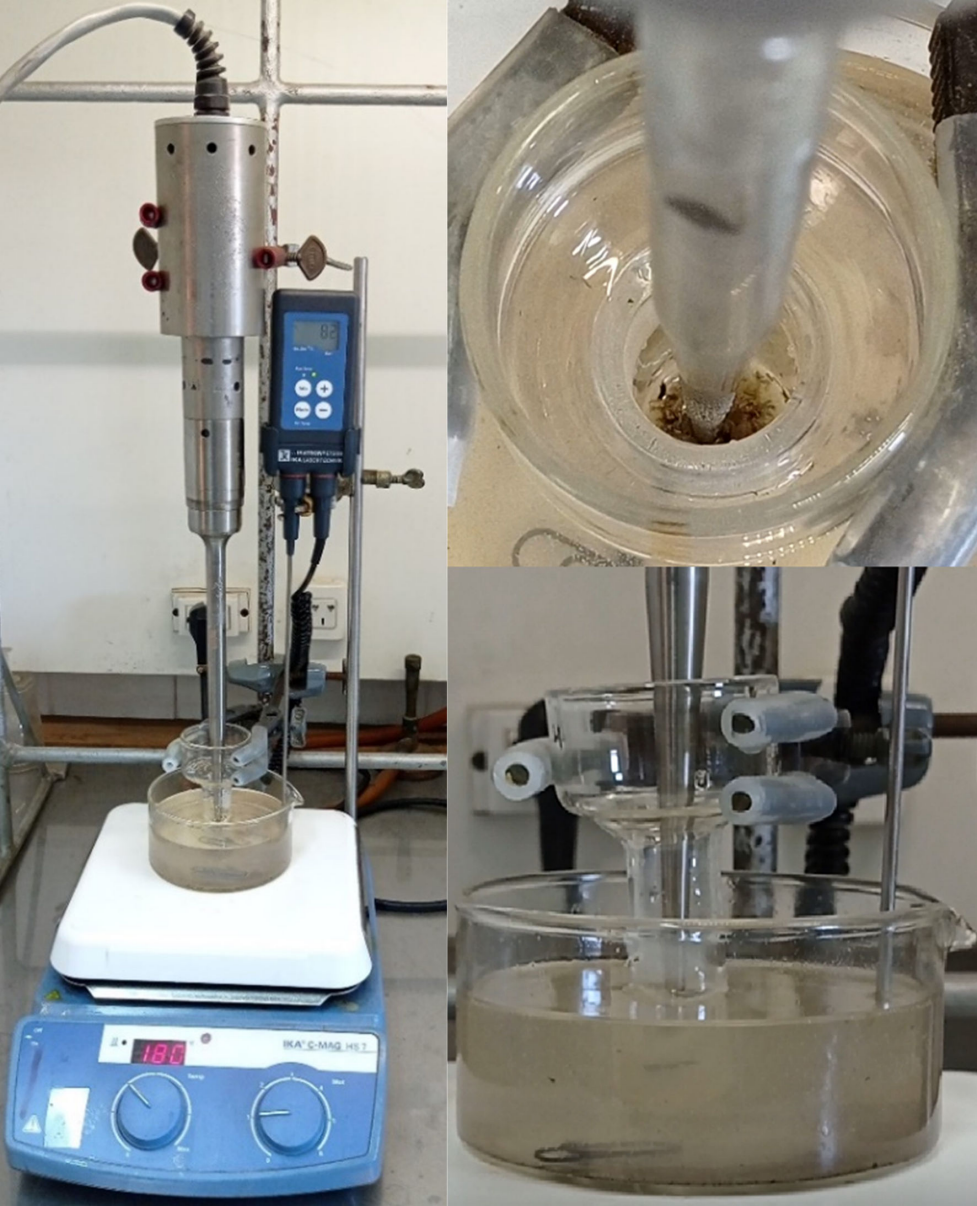
Arrangement of the probe inside the glass container with the sample.

Based on the above, we decided to work with whole sunflower husks, imidazolium salts and US. Aqueous solutions of known concentrations of the salts (0.5% or 2.5%) were prepared, into which the whole sunflower husks were incorporated and subjected to US using an ultrasonic probe. The operational conditions are detailed in table 1. For sonication, maximum, intermediate and minimum settings (4, 2.5 and 1, respectively) were used, with a sonication time of 5 min and a constant temperature of 80°C. The reaction medium included salt at concentrations of 0.5% and 2.5%, and 50 mg of husks in each experiment. Figure 1 shows the placement of the ultrasonic probe inside the glass container containing the sample.

**Table 1.** Experimental conditions. (Aqueous solution (2 ml) of imidazolium salt, 50 mg of shells. The mixture was exposed to ultrasonic irradiation at 80°C in a water bath for 5 min.)

entry	salt	conc (%)	power (US)	duty cycle (% US)
1	—	—	—	—
2	—	—	4	80
3	—	—	1	10
4	—	—	2.5	50
5	**S1**	0.5	4	80
6		2.5	4	80
7		0.5	1	10
8		2.5	1	10
9		2.5	2.5	50
10	**S2**	0.5	4	80
11		2.5	4	80
12		0.5	1	10
13		2.5	1	10
14		2.5	2.5	50
15	**S3**	0.5	4	80
16		2.5	4	80
17		0.5	1	10
18		2.5	1	10
19		2.5	2.5	50

### 2.4. Biological test

Three replicates were performed for each experiment. One husk was placed per well in a cell culture plate, which was then inoculated with *S. albus* CAS922 and incubated at 37°C for 3 days. Following incubation, bacterial colony growth was assessed. The husks were individually removed from the plate and transferred to Eppendorf tubes containing physiological solution. Each tube was vortexed for 1 min. A 20 μl aliquot of the physiological solution was then diluted to 180 μl (dilution 1), and this process was repeated two more times (dilutions 2 and 3). From each dilution, 20 μl was plated on agar plates with culture broth and incubated at 37°C for 2 days. After incubation, the bacterial colonies were counted (see the electronic supplementary material).

## 3. Results and discussion

### 3.1. Sulfonated-imidazolium salts

#### 3.1.1. Synthesis

**Figure 2.**
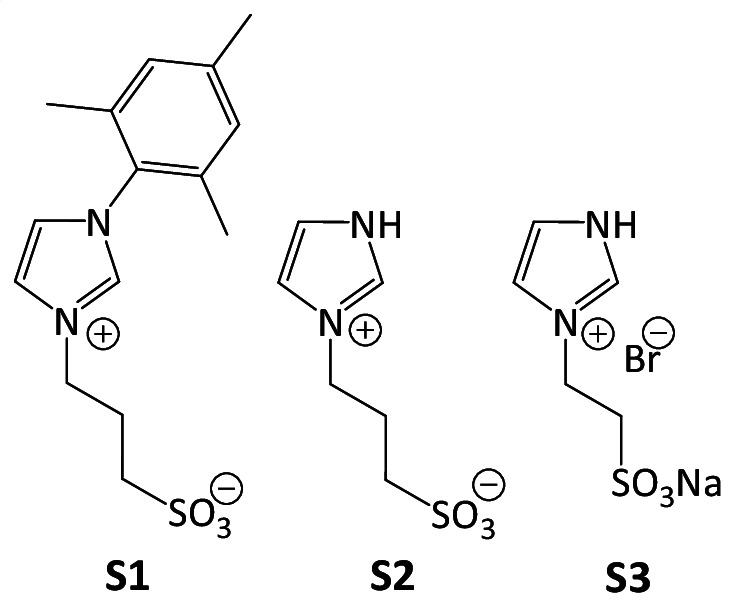
Imidazolium salts used.

Three imidazole salts (**S1**, **S2** and **S3**; see figure 2) were synthesized via a quaternization reaction. **S1** was synthesized from N-(2,4,6-trimethylphenyl)imidazole, while salts **S2** and **S3** were synthesized from imidazole. All salts, obtained as white solid, contain a sulfonate group, which enhances their solubility in water. Salts **S1** (98% yield) and **S2** (99% yield) contain a propylsulfonate group, whereas **S3** (85% yield) contains an ethylsulfonate group. Additionally, **S1** features a bulky mesityl group while **S2** and **S3** have a proton. These variations were selected to investigate the effects of different substituents and steric bulk on the treatment of lignocellulosic residue.

#### 3.1.2. Nuclear magnetic resonance characterization

*1-Mesityl-3-(3-sulfonatopropyl)imidazolium* (**S1**). ^1^H NMR (300 MHz, D_2_O): δ 8.93 (s, 1H, NCHN), 7.75 (s, 1H, Imz, Ar C4H), 7.51 (s, 1H, Imz, Ar C5H), 7.08 (s, 2H, Ar), 4.44 (t, 2H, NCH_2_); 2.91−2.89 (m, 2H, SCH_2_), 2.37- 2.33 (m,2H, CH_2_CH_2_CH_2_), 2.27 (s, 3H, *p*-MeAr), 1.96 (s, 6H, *o*-MeAr); ^1^H NMR (300 MHz, DMSO-d_6_): δ 9.26 (s, 1H, NCHN), 7.98 (s, 1H, Imz, C4H), 7.78 (s, 1H, Imz, C5H), 7.00 (s, 2H, Ar), 4.30 (t, 2H, NCH_2_), 2.36−2.29 (m, 2H, SCH_2_), 2.19 (s, 3H, *p*-MeAr), 2.15–2.00 (m, 2H, CH_2_CH_2_CH_2_), 1.89 (s, 6H, *o*-MeAr); ^13^C NMR (75 MHz, D_2_O): δ 141.3 (s, Ar C1), 136.5 (s, Imz C2), 134.6 (s, Ar C2), 130.7 (s, Ar C4), 129.1 (s, Ar C3), 124.3 (s, Imz C4), 122.9 (s, Imz C5), 48.1 (s, NCH_2_), 47.13 (s, SCH_2_), 24.9 (*p*-MeAr), 20.1 (s, CH_2_CH_2_CH_2_), 16.2 (s, *o*-MeAr); ^13^C NMR (75 MHz, DMSO-d_6_): δ 140.1 (s, Ar C1), 137.5 (s, Imz C2), 134.3 (s, Ar C2), 131.1 (s, Ar C4), 129.2 (s, Ar C3), 123.8 (s, Imz C4), 123.2 (s, Imz C5), 48.3 (s, NCH_2_), 47.3 (s, SCH_2_), 26.0 (*p*-MeAr), 20.5 (s, CH_2_CH_2_CH_2_), 16.8 (s, *o*-MeAr). (*3-sulfonatepropyl)imidazolium* (**S2**). ^1^H NMR (300 MHz, D_2_O): δ 8.79 (s, 1H, NCHN), 7.59 (s, 1H, Imz, C5H), 7.51 (s, 1H, Imz, C4H), 4.44 (t, 2H, NCH_2_), 2.96 (t, 2H, SCH_2_); 2.42−2.32 (m, 2H, CH_2_CH_2_CH_2_); ^1^H NMR (300 MHz, DMSO-d_6_): δ 14.24 (s, 1H, NH), 9.11 (t, 1H, NCHN), 7.79 (t, 1H, Imz C5H), 7.68 (t, 1H, C4H), 4.32 (t, 2H, NCH_2_), 2.40 (t, 2H, SCH_2_), 2.10 (q, 2H, CH_2_CH_2_CH_2_); ^13^C NMR (75 MHz, D_2_O): δ 134.7 (s, Imz C2), 121.8 (s, Imz C4), 119.9 (Imz C5), 48.0 (s, NCH_2_), 47.6 (SCH_2_), 25.1 (CH_2_CH_2_CH_2_); ^13^C NMR (75 MHz, DMSO-d_6_): δ 135.4 (Imz C2), 122.1 (Imz C4), 119.9 (Imz C5), 47.5 (NCH_2_), 47.4 (SCH_2_), 26.2 (CH_2_CH_2_CH_2_). (*3-Sodiumsulfonatoethyl)imidazolium bromide* (**S3**). ^1^H NMR (300 MHz, D_2_O): d 8.68 (s, 1H, NCHN), 7.50 (s, 1H, Imz, C5H), 7.38 (s, 1H, Imz, C4H), 4.57 (t, ^3^*J*_H-H_ = 7.0, 2H, NCH_2_), 3.38 (t, ^3^*J*_H-H_ = 7.5, 2H, CH_2_S); ^1^H NMR (300 MHz, DMSO-d_6_): d 9.04 (s, 1H, NCHN), 7.78 (s, 1H, Imz, C5H), 7.59 (s, 1H, Imz, C4H), 4.41 (t, ^3^*J*_H-H_ = 7.0, 2H, NCH_2_), 3.02 (t, ^3^*J*_H-H_ = 7.5, 2H, CH_2_S); ^13^C NMR (75 MHz, D_2_O): d 135.4 (s, Imz C2), 121.8 (s, Imz C5), 120.2 (s, Imz C4), 49.9 (s, NCH2), 44.8 (s, SCH2); ^13^C NMR (75 MHz, DMSO-d_6_): d 136.4 (s, Imz C2), 122.7 (s, Imz C5), 120.3 (s, Imz C4), 46.4 (s, NCH_2_), 46.1 (s, SCH_2_).

### 3.2. Ultraviolet–visible spectroscopy

**Figure 3.**
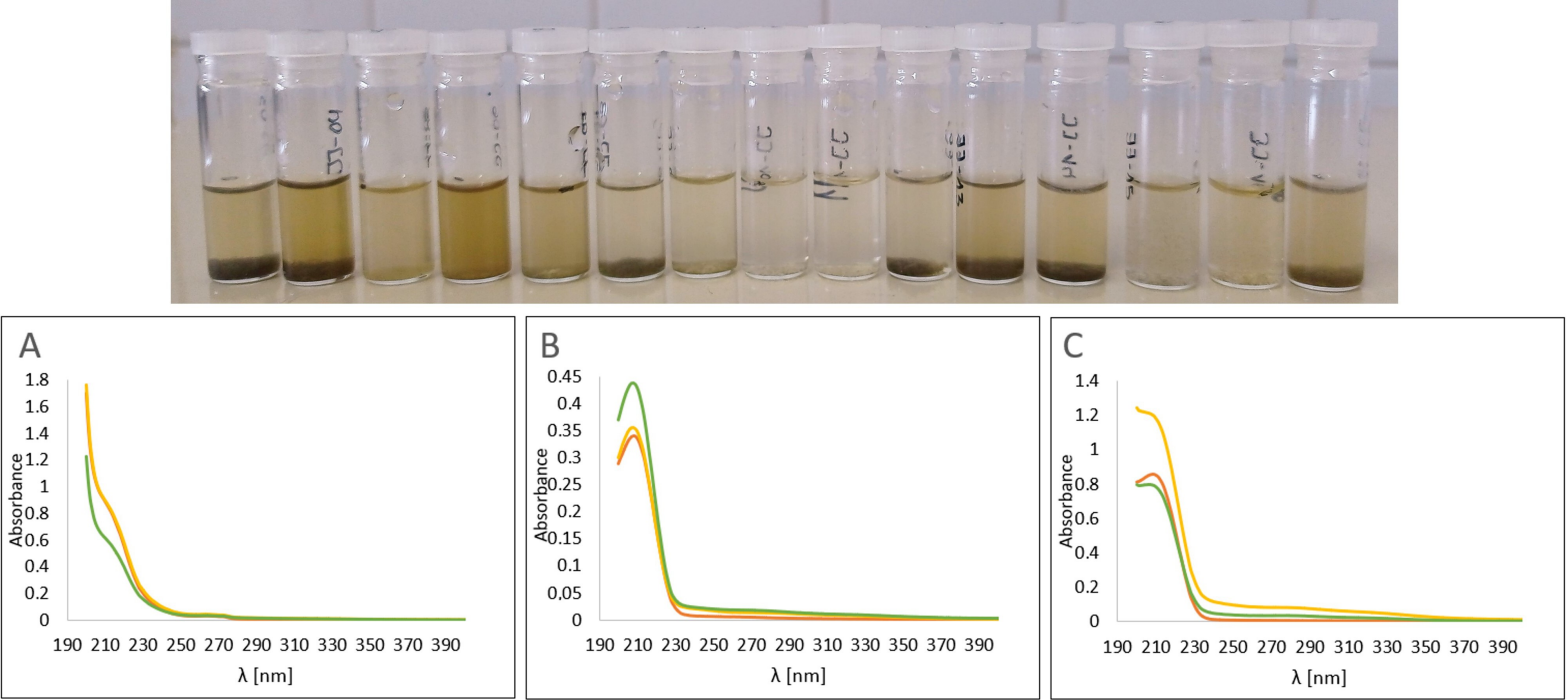
Aqueous solutions of the salts after sonication and their corresponding UV–Vis spectra: (A) experiments 5 and 7; (B) experiments 10 and 12; and (C) experiments 15 and 17.

After each treatment, the resulting solution consistently exhibited a brownish colour and, in some cases, suspended solids (see figure 3), attributed to residues from the sunflower husks. UV–Vis spectroscopy was employed to detect the presence of the salt post-treatment. Spectra were recorded both before treatment (blank) and after treatment for experiments with a salt concentration of 0.5% (refer to figure 3A: entries 5 and 7; figure 3B: entries 10 and 12 and figure 3C: entries 15 and 17, respectively). As observed from the spectra, there is no modification or loss of the salt during ultrasonic treatment.

**Figure 4.**
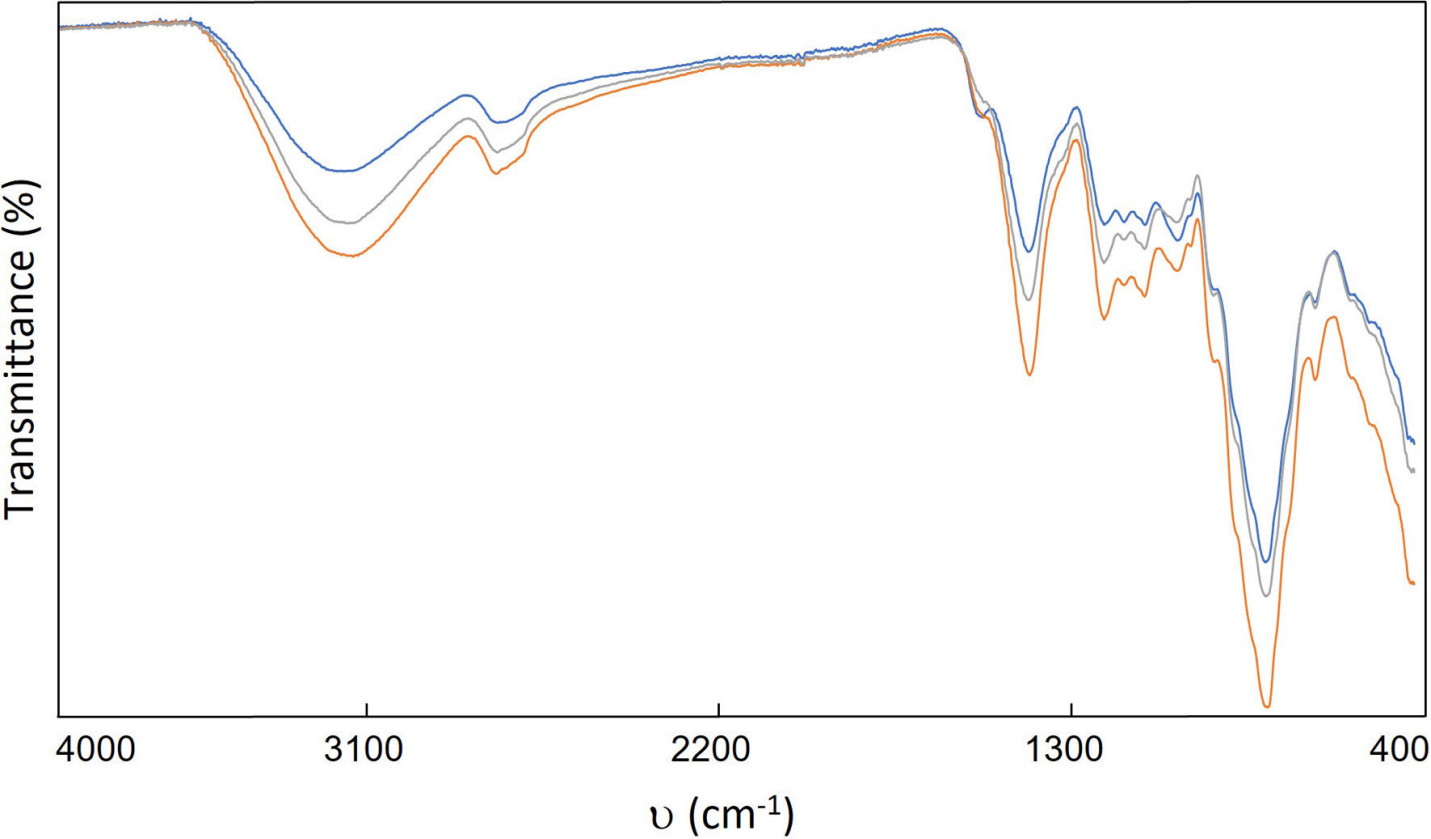
ATR-FTIR spectrum of treated sunflower hulls (table 1, entry 5).

On the other hand, the sunflower husks were analysed using ATR-FTIR spectroscopy to determine whether the functional groups of the husk components were altered by the salts or US. Three samples from each experiment were examined, and readings were taken for each. Figure 4 shows the spectrum corresponding to entry 5 (table 1). A similar trend was observed across all experiments, indicating that the functional groups of the sunflower husks do not undergo significant changes (see the electronic supplementary material).

### 3.3. Biological test

**Figure 5.**
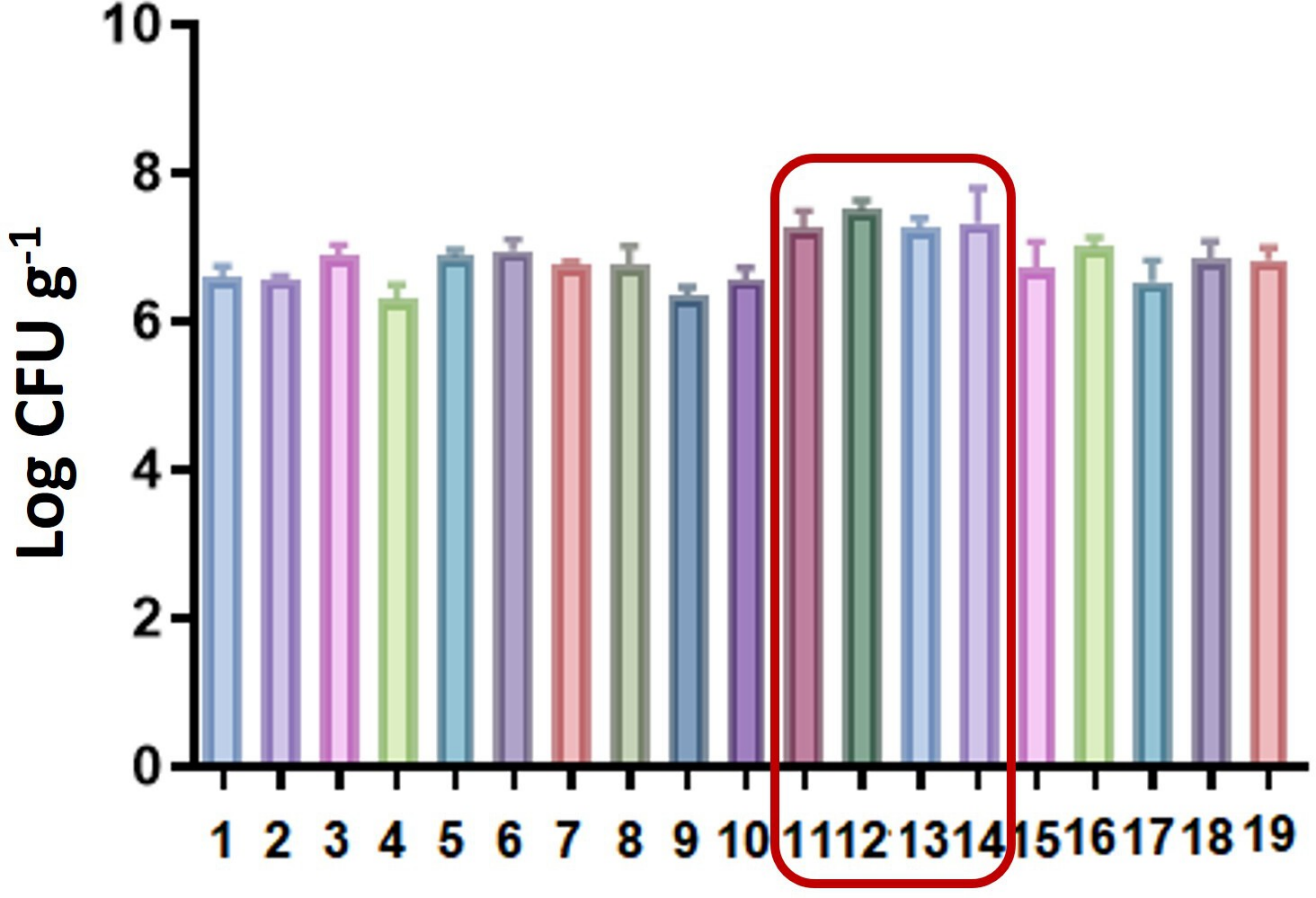
Bar chart from the Dunnett test comparing experiments to entry 1 (table 1).

After treatment, the sunflower husks were subjected to a biological assay using a *S. albus* CAS922 culture. The obtained data were analysed by plate counting to calculate the average number of colony-forming units (CFU) per gram of husk. Statistical analyses, including ANOVA and Dunnett’s test, were conducted to compare the results of the experiments against entry 1 (table 1) as a control. It was found that entries 11 to 14 showed significant differences (*p* < 0.05, see figure 5).

Entries 11−14, which involved salt **S2** under varying US conditions and concentrations, showed statistically significant effects, suggesting a combined influence of salt concentration and sonication parameters. Notably, entries 10 and 12 both used 0.5% **S2** but differed in US settings, while entries 11, 13 and 14 used higher salt concentrations (2.5%) with different US combinations. Additionally, entries 5, 10 and 15, which shared the same US parameters but differed in salt type (**S1**, **S2** and **S3**, respectively), highlight the key role of salt structure in determining the best growth conditions for the microorganism of interest. These results demonstrate that both the chemical nature of the salt and the processing conditions may be critical factors in optimizing the growth of the producing microorganism in treated sunflower husks.

**Figure 6.**
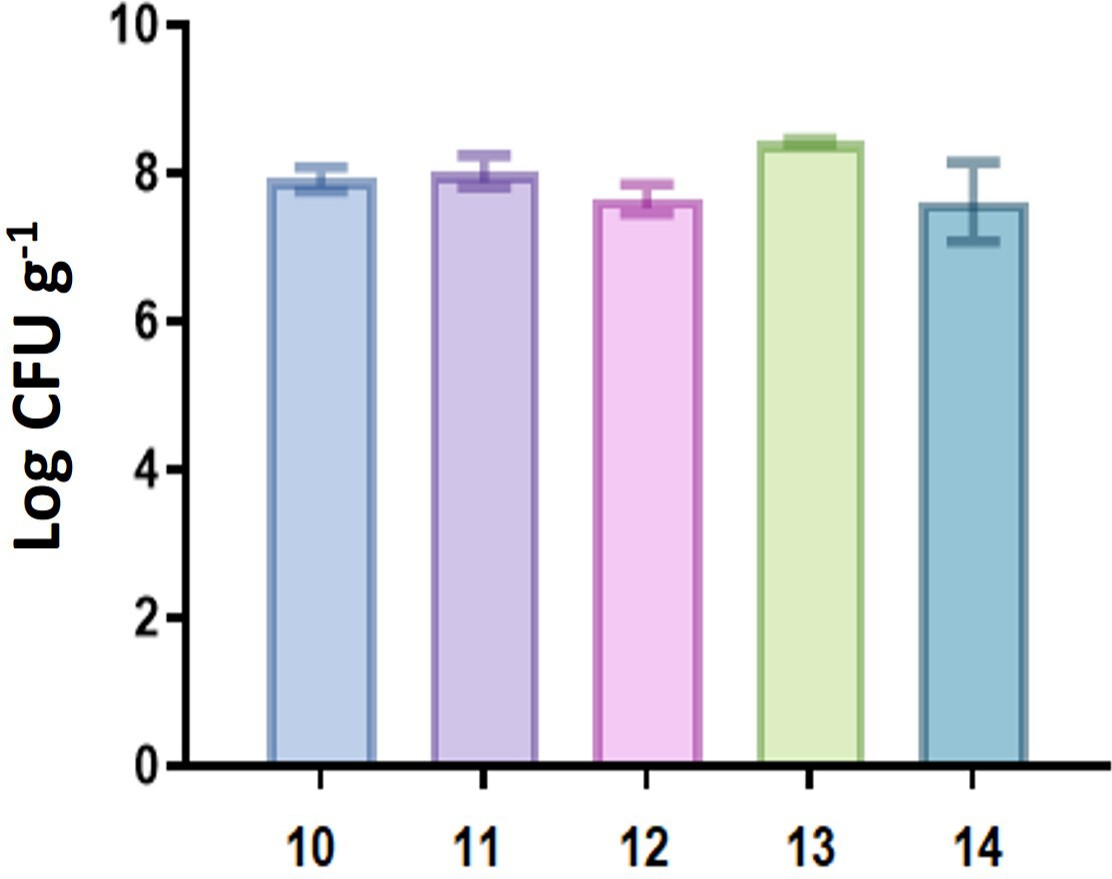
Tukey test bar chart.

After establishing that salt **S2** had a significantly superior effect on the shell compared to the other two salts, we conducted further tests exclusively with **S2** to identify the most effective instrumental conditions. We applied the previously described treatment using imidazolium salt solutions and US and repeated the bioassay in quintuplicate, including the corresponding dilutions. Following the incubation period, we performed colony counting. The results of the statistical analyses (ANOVA and Tukey) revealed that entry 13 (table 1) achieved a slightly better performance than the other experimental conditions (*p* < 0.05, figure 6). This convergence in results suggests that the growth=promoting effect may plateau beyond a certain threshold or be influenced by experimental variability, thereby masking small differences under repeated conditions. The results indicate that bacteria can thrive on treated sunflower hulls, with some cases showing even enhanced growth. This presents an opportunity for cultivating other bacteria that can convert waste into industrially valuable molecules.

### 3.4. Salt reuse study

**Figure 7.**
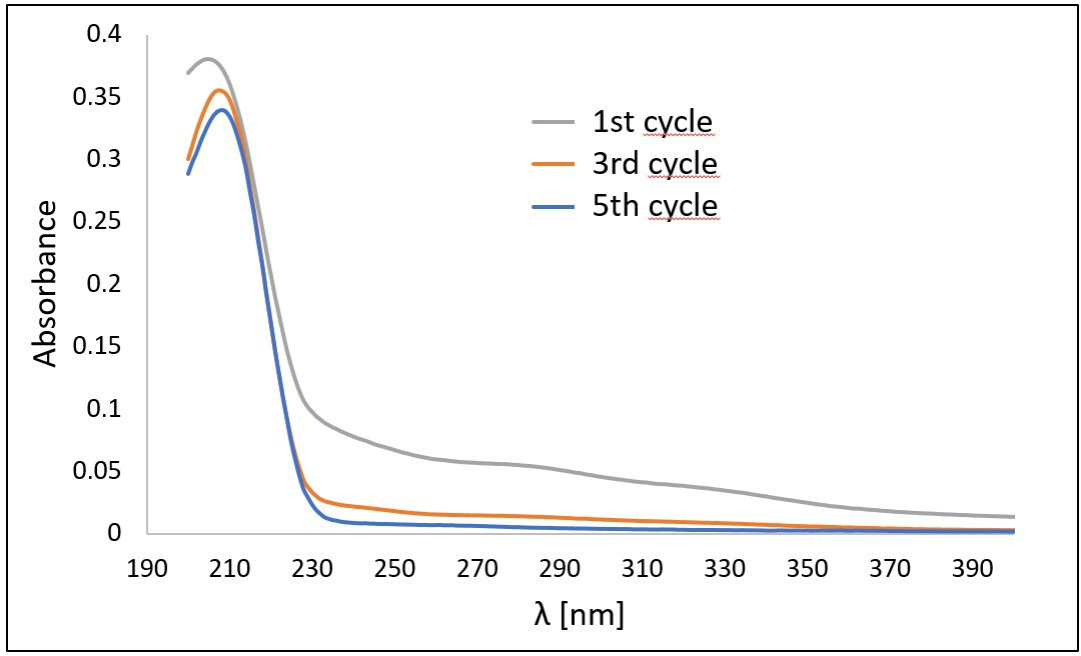
UV–Vis spectra of salt **S2** after one, three and five cycles, respectively.

The reuse of **S2** salt was examined at a concentration of 0.5% using the instrumental conditions outlined in entry 13 (table 1, power/duty cycle 1/10). A solution was prepared, and UV–Vis spectra were recorded before subjecting the solution to five cycles of US treatment. After each cycle, the shells were removed and replaced with new ones. Readings were taken after three and five cycles. As shown in figure 7, there is no appreciable change in the shape of the band after five cycles of sonication, indicating the stability of the salt under the applied conditions.

## 4. Conclusion

The desired salts were synthesized and characterized, yielding results similar to those reported in the literature. The treatment conditions (temperature and US) did not modify the chemical structure of the lignocellulosic residue, as observed by FTIR. While US does not modify the functional groups, as observed by FTIR, the rarefaction cycles induce fibre and cell rupture (cell lysis) [4] This disruption facilitates bacterial growth by making it easier for bacteria to break down the shell components. The study on the reuse of aqueous solutions demonstrated that **S2** salt can be reused effectively for up to five consecutive cycles, provided the lignocellulosic residue is changed. No loss of salt concentration was observed, indicating that **S2** exhibits strong stability against both temperature and US. In summary, advanced conditioning of lignocellulosic wastes using imidazolium salts and US energy offers a promising opportunity to cultivate additional bacteria capable of converting waste into valuable industrial molecules.

## Data Availability

The datasets supporting this article have been uploaded as part of the supplementary material [27].
